# Beyond the Present Constraints That Prevent a Wide Spread of Tissue Engineering and Regenerative Medicine Approaches

**DOI:** 10.3389/fbioe.2019.00095

**Published:** 2019-05-07

**Authors:** Benjamen T. O'Donnell, Clara J. Ives, Omair A. Mohiuddin, Bruce A. Bunnell

**Affiliations:** ^1^Center for Stem Cell Research and Regenerative Medicine, Tulane University School of Medicine, New Orleans, LA, United States; ^2^Department of Pharmacology, Tulane University School of Medicine, New Orleans, LA, United States

**Keywords:** regenerative medicine, tissue engineering, regulation, food and drug administration, translation, commercialization

## Abstract

Despite the success of tissue engineered medical products (TEMPs) in preclinical translational research, very few have had success in the clinical market place. This gap, referred to as the “valley of death” is due to the large number of ventures that failed to attract or retain investor funding, promotion, and clinical acceptance of their products. This loss can be attributed to a focus on a bench to bedside flow of ideas and technology, which does not account for the multitude of adoption, commercial, and regulatory constraints. The implementation of an alternative bedside to bench and back again approach permits investigators to focus on a specific unmet clinical need, defining crucial translation related questions early in the research process. Investigators often fail to accurately identify critical clinical adoption criteria due to their focus on improved patient outcomes. Other adoption criteria (such as price, time, ethical concerns, and place in the workflow) can cause a product to fail despite improved patient outcomes. By applying simplified business principles such as the build-measure-learn loop and the business model canvas to early-stage research projects, investigators can narrow in on appropriate research topics and define design constraints. Additionally, 86% of all clinical trials fail to result in Federal Drug Administration approval, resulting in significant economic burdens. On the reverse side, approval through the European Medical Agency is widely considered to be more direct but has its challenges. The Committee for Advanced Therapies within the European Medical Agency has received 22 market authorization applications for advanced therapy medicinal products, of which only 10 received authorization. A thorough understanding of the various regulatory pathways permits investigators to plan for future regulatory obstacles and potentially increase their chances of success. By utilizing a bedside to bench and back again approach, investigators can improve the odds that their research will have a meaningful clinical impact.

## Introduction

The last two decades have seen several successful clinical applications of tissue engineered medical products (TEMPs). Autologous cell-based therapies, such as Carticel (which utilizes autologous chondrocytes to repair focal articular cartilage defects), have been the most successful (Dewan et al., 2014). Decellularized scaffolds, such as Dermapure, have also been successful in wound healing applications (Otto et al., 2016). The success of these products is a result of concentrated federal funding into the tissue engineering field. The National Institutes of Health (NIH) of the United States alone has invested an estimated $940 million in 2017 on regenerative medicine research^1^. That funding has resulted in numerous successful research projects and published works, as reviewed by Park et al. (2018).

Despite the success of a handful of companies and the overabundance of research, TEMPs still have not yet reached mainstream application (Mao and Mooney, 2015). TEMPs are limited by their inability to effectively recapitulate the complex cellular, structural, and mechanical environment of native tissues when transitioning from *in vitro* to *in vivo* applications. The high failure rate is in part attributed to a focus on benchtop success before undertaking translational studies in humans. This method often fails to account for the criteria that determine the translational success and lifespan of a product. Therefore, in order to improve clinical success, investigators need to look beyond the present scientific constraints that limit clinical translation of TEMPs and critically analyze the methodology that guides the translational application of TEMPs. The crucial component is the development of an interactive bedside to benchtop and back again approach that allows investigators to understand future translation hurdles.

Development of bedside to bench and back again approach can possess challenges that most investigators are not typically trained to overcome. Regulatory, commercialization and adoption challenges require foresight and proper planning to navigate, and it is often too late in the developmental process to make substantive changes to TEMPs when these challenges arise. Investigators who wish to see their products reach the clinical market should institute essential business and regulatory methodology early on in development while the product is still at the bench to increase the likely hood for translation.

In this commentary, several key criteria that should be considered by researchers developing TEMPs are discussed, including clinical adoption, commercialization, and governmental regulation.

## Considerations for Clinical Adoption

Translation to clinical practice bridges the gap between benchtop invention and marketable technology. This gap is commonly termed the “Valley of Death” due to the large number of ventures that quickly lose interest from investors, promotion, and adoption for clinical use due to unanticipated product limitations (Fernandez-Moure, 2016). Maintaining clinical and investor support is particularly onerous because TEMPs require potentially high upfront costs for development, challenges concerning funding for large-scale preclinical and clinical studies to gain approval by regulatory bodies, demonstrating product safety, and fostering clinical endorsement.

### Design Constraints

During the initial development and characterization of a TEMP, the *in vitro testing* via cell culture and *in vivo* assessment in animal models need to be guided by the clinical application endpoint. The physical, mechanical, chemical, and degradation properties that comprise the design criteria, as well as biocompatibility and reproducibility, should be routinely weighed against current medical solutions. Physicians tend to be “creatures of habit,” and the physician assessment of potential risk is an imposing impediment for the clinical adoption of TEMPs and cause for clinical conservatism with new products. Physician acceptance will depend primarily on how the proposed TEMP improves upon existing clinical approaches, evidenced by better patient outcomes, reduced cost and operating time, and pragmatic integration into operating room workflow (Hollister and Murphy, 2011). Furthermore, from the physician's perspective, a product must be ergonomic, straightforward, and efficient to handle and apply, as new products are often met with hesitation if they require uncomfortable or non-standard delivery mechanisms or require more time to implant and maintain (Dlaska et al., 2015). Thus, the culture change of physicians is driven chiefly by a large body of evidence of safety assurance and the TEMPs ability to fit into the current procedures of the operating room. Early involvement of surgeons and physicians in the TEMP design process can expose them to the necessary skills and techniques and allow developers to address pragmatic concerns and recognize potential regulatory complexities. TEMPs have yet to be widely applied in a clinical setting, resulting in skepticism that deems even the most straightforward or most conventional products as fundamentally radical.

In the TEMP product landscape, cell-free native tissue scaffolding is relatively simple and amenable to scale up in manufacturing with little risk of lot-to-lot variability and quality control liability. Also, immune rejection of cell-free scaffolds is unlikely. In contrast to their acellular counterparts, cell-integrated TEMP products often pose unique design and manufacturing complications and are categorized by their cell source: (1) autologous cells harvested from a patient tissue, (2) donor-derived allogeneic cells, and (3) cross-species transplanted xenogeneic cells. While cell-based products will possess some level of risk for immune rejection, the appropriate design controls regarding cell sourcing, processing, and characterization can limit immunogenic uncertainty. Broadly, autologous cells do not require immunosuppressive therapy upon implantation due to their inherent *in vivo* compatibility and thus, have potential in personalized medicine. However, they require additional procedures for tissue harvesting which can lead to secondary site pain and have no guarantee of *in vitro* reproducibility and viability. New engineering strategies show promise for alternative non-native cell types, including modification of allogeneic and xenogeneic cells to reduce immunogenic potential (Hellman, 2006). Good manufacturing practice (GMP) standards must be maintained for any product. Further considerations should be given to scaling cellular products for manufacturing. Several key issues include culture space requirements, the stability of cellular phenotypes, and methods for cellular delivery and treatment localization. Maintaining the cells as a homogenous suspension will guide downstream processing and purification. TEMPs often integrate multiple biologics; the combination of cells, chemicals, and biomaterials complicates process control. Process monitoring for scale-up manufacturing is critical to assure stable and reproducible product; these systems include quality control assays, tissue-customized bioreactors and *in vitro* modeling and simulation, and immunogenicity testing for both local and systemic responses (Hellman, 2006; Webber et al., 2015).

Given the variability of host tissue integrity, anatomic loading conditions, and the cellular microbiome, the complex dynamics of *in vivo* tissue environment are challenging to normalize (Webber et al., 2015). Numerous companies have increased product complexity and developed highly convoluted solutions in an attempt to accommodate these mechanical and chemical cues. Realistically, the development of TEMPs with multiple materials for both deliveries of biologics and structural support is virtually impossible within a simple, single-step fabrication process. While complex therapies have potential to infiltrate larger markets, they are met with significant risks, increased regulation, considerable R&D and quality system costs, and longer development time (Gelijns et al., 1990). The inherent challenges of product complexity, however, may be mitigated by a modular approach. Modular TEMP designs have components that may be individually tuned for easy adoption by other clinical markets. Modularity allows for the separate implementation of design control and quality processes of each facet, thereby simplifying translation to large-scale production, which is essential to making scaffolds practical and cost-effective as viable clinical approaches. The ability to troubleshoot individual components of the product or fabrication process simplifies problem-solving and reduces expenses, which is a significant advantage leading into clinical trials (Hollister and Murphy, 2011; Vincent et al., 2014).

### Value Proposition

After initial product development, the uniqueness and utility in the context of specific customer needs provide a compelling and novel value proposition. An excellent candidate for successful commercialization is a platform technology, meaning it has the potential to be used across multiple applications. A powerful technology that only has niche applicability has a reduced likelihood for success than one that could result in products in different areas (Webber et al., 2015). The value proposition is the hypothesized way that a company will appeal to its target market, developed after careful review of the costs and benefits a device can provide for its customers and other constituent groups. As the needs of the market prove different from expectations, the matter of strategic change through needs-based thinking, such as value proposition evolution, is critical as ventures develop. Companies are known to fail when the market demands product evolution and the need is recognized, but adaptation is constrained by the inherent parameters of the specific technology (Covin et al., 2015). It is highly recommended that start-up companies pursue a clearly defined market with a robust value proposition. For this, niche markets are often ideal candidates because they are readily defined, their value proposition are clear, and they are easier to capture. However, it is essential for entrepreneurs to realize that, in general, a single product cannot support a company. Entrepreneurs need to plan for multiple potential markets and uses after the success of their original product. Each of these markets will have a different value proposition and flexibility can allow for multiple applications of the same product. All sectors of financial support (government, private, and public institutions) consider TEMPs to be challenging to evaluate due to the poorly established regulatory and clinical pathways, and so regenerative medicine representatives seeking capital from the financial industry must keep the description of their technology and its value, above all else, direct and straightforward.

### Ethical Considerations

Ethical concerns regarding the use and distribution of tissues and cells for TEMPs has been a source of debate; these issues can be classified by (1) the source of tissues and cells, (2) the donation process, and (3) the manipulation of cells to generate the TEMP (de Vries et al., 2008). The primary source of human embryonic stem cells and fetal cells is aborted fetal tissue—in itself, a highly controversial moral intervention—as the use of fetal tissue from elective abortion is seen by many as a way of legitimizing abortion. Thus, the dispute over isolation of stem/progenitor cells from these sources remains highly divisive. Many companies attempt to avoid this issue through the use of adult stem cells in their TEMP products. Adult stem cells have fewer ethical concerns but have increased cellular variability. As to tissue donation, informed consent must, at an absolute minimum, constitute a voluntary decision based on full disclosure of information. Consent for donated tissue is paramount; donors must be informed about future applications and use of their cells, tissues or organs, and the anonymization of donated samples must be maintained to protect donor privacy and avoid exploitation of vulnerable participants (Otto et al., 2016). A closely related issue is the question of ownership. Controversy over donated human cells arises from the question of whether human tissue is subject to laws regarding property rights. Additionally, objections to therapeutic cloning, genetic engineering, and the merging of human and animal cells have been raised (Taylor et al., 2014). Limitations imposed on ethical grounds for the acquisition and distribution of human and animal cells, therefore, must be recognized and navigated with consideration and discretion.

Another ethical quandary in the development of any novel therapeutic, the “right to try” law, stems from the Compassionate Care Act and allows terminally-ill patients to seek treatment using products that have not completed the full regulatory approval process. The burdens and risks to participants at this early stage in development must be weighed against anticipated benefits (Otto et al., 2016). While early clinical research has the potential to validate product efficacy, early trials can also compromise patient health and damage public perception, although onlookers should be able to recognize the inadequacy of conventional treatments and the likelihood of co-morbidities. Regardless, this form of clinical translation requires thorough ethical reflection.

Related to the “right to try” is the responsibility of companies to ensure their new TEMP does not harm the patient. The TEMP industry has been plagued, like many other medical industries, with the high-profile failure of TEMP that were not ready for the market. The U.S. is currently facing an intense debate over the ethical use of autologous stem cells therapies. In 2014, several clinics utilized adipose-derived mesenchymal stem cell injections into patients eyes in an attempt to combat macular degeneration (McGinley and Wan, 2019). However, after several patients went blind, those clinics are still offering those treatments and the U.S. government has had difficulty stopping them (McGinley and Wan, 2019). Although autologous stem cell has the potential to be a very powerful therapeutic, deeper investigation is required to determine the risk factors. Failures like this one are often widely covered, creating fear and paranoia toward products that have been properly investigated. It is the ethical responsibility of investigators and clinicians to ensure the proper use of TEMP, both for the good of the product and the industry.

## Commercialization

It is a common misconception among researchers that in order to spin out a product from their laboratory, it must encompass a finalized and complete product, ready for human clinical trials. In actuality, the vast majority of research projects are in the early stages of their development and are not ready for immediate clinical application. By implementing recently developed entrepreneurial principles in the early stages of TEMP development, investigators can shrink the gap between their bench and the patient.

The publication of books such as *The Lean Startup* by Eric Reis and the business model canvas by Alexander Osterwalder has revolutionized the way that entrepreneurs and investors approach building businesses, changing the criteria by which they base their value of products along the way (Ries, 2011). It is more important than ever for researchers who wish for their projects to reach the clinical market, either through a startup or license to an established company, to correctly identify the value of their projects in the early stages and allow for market needs to help shape the path of their research.

### A Lean Benchtop

*The Lean Startup* is a renowned book that describes applying the scientific methodology to startups, using examples from software startups. In the book, Reis describes a Build-Measure-Learn loop (BML) as a way of designing products using customer input. Although the BML model is often criticized for difficulty in translation to the TEMP field, there are multiple methodologies within it that investigators can apply early-on to research that will allow them to avoid obstacles. The minimum-valued-product (MVP) is the product that includes its most essential functions, as hypothesized by the inventors, but is not necessarily a totally streamlined product. The MVP can be released to a sample group of customers, who provide feedback that either validates the inventor's hypothesis or prompts them to pivot and redesign their product. An MVP cannot be applied for use in human clinical trials because the system is too inflexible and expensive. However, investigators can and should employ the MVP early on their experimental process, especially in the *in-vitro* and *in-vivo* models. The crucial step here is to avoid the bench to bedside approach where an investigator has developed a product and begins to search for a relevant market, but instead has defined a clinical need and begins to develop a TEMP to address that need. This bedside to bench and back again approach allows researchers to define clinically essential criteria in early hypotheses and then utilize the BML to shed extraneous experiments and focus the research on developing a product. A drawback to this approach is that many investigators are not trained to do market research as academia, including the grant funding processes, rarely address these skills. As such, there have been several state and federal programs developed over the past several years the focus of which is to enhance the translation of academic research to the market place.

### The Business Model Canvas and the I-Corps Program

**Figure 1:**
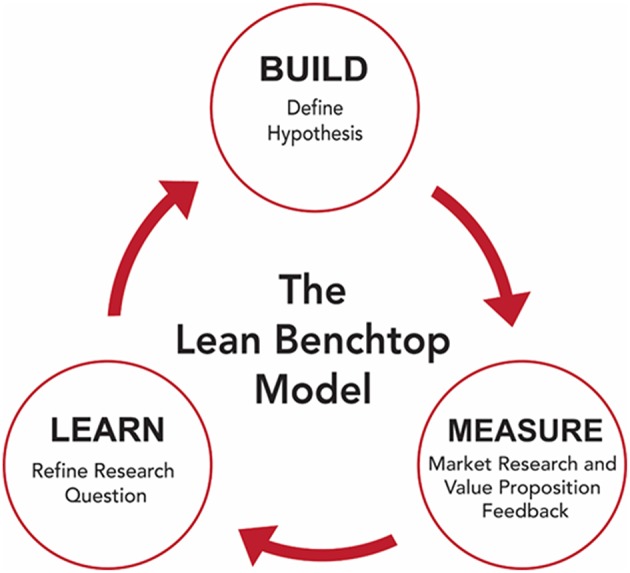
The build-measure-learn loop utilized to create the lean benchtop model.

Osterwalder originally proposed the business model canvas (BMC) in 2008, and over the past 10 years, has grown and developed into a useful tool often applied before founding a company (Osterwalder, 2008). Sometimes described as a business hypothesis, the BMC was designed to permit entrepreneurs to apply their concept to a market segment rapidly. Within the BMC, entrepreneurs define the value proposition, customer segments, key activities/products/resources, customer relationships, channels, cost structure, and revenue streams. It is common practice to fill out the BMC many times for one given idea, exploring the different directions the company can go. From there, entrepreneurs will determine the central hypotheses that each version of the company would be built upon, which can then be validated before the business is founded. For academic entrepreneurs, the BMC represents a valuable tool that will help guide the inexperienced through the first crucial steps of their company and answer the question “do I have something of value here?” Figure 1 illustrates the combination of using the BML to investigate the market segment of the BMC. By validating their goals in the same way they would conduct their research, investigators can improve their clinical impact and streamline their research process. The BMC will also highlight weaknesses within the startup team and is an opportunity to bring new people onto the team that can address them.

I-Corps, initially developed by the National Science Foundation (NSF) in the US, has both regional and national program components. The express goal of the program is to increase the translation of research to products, a gap identified by Congress who began asking the questions of how research dollars directly benefits the taxpayers who provide them. The I-Corps program focuses on the notion of teaching investigators how to complete market research and providing incentive funds that allow them to leave the lab to execute the market analysis. The programs highlight four components of the BMC; the value proposition, the customer segment, customer relations, and the channels upon which their product will reach their customer. Regional programs usually include small incentives, $1,000–$3,000, and include doing 10–30 interviews. The national I-Corps requires either funding by an NSF grant or participation in a regional I-Corps program. National I-Corps participants are required to conduct over 100 interviews and receive $50,000 to use for travel related to interviews and mentoring sessions^2^. The interviews are meant to cover all parts of the target customer segment, not only the direct end users, which is a vital step for most TEMPs because they often have a complex customer composition that includes not only the patient, but also doctors who diagnose and implement the product, health insurance providers who pay for the product and procedure, and even hospital staff who decide what products are used in the clinic. Most often, participants in I-Corps find that the assumptions about their customer base are false, especially concerning who their customer will be and the value their product brings. Customer interviews allow investigators to pivot into a new direction before their company hits an obstacle from which it cannot recover. Additionally, many regional I-Corps programs include lectures and training sessions for busy investigators who are unsure of investing the time and effort into a formalized program. Recently, the I-Corps program has been expanded to the NIH, expanding the opportunities for many investigators. While there are multiple grant opportunities offered by the European Union (EU) for translational research, programs similar to the I-Corps program could not be identified.

### The Spinout Team

The creation of a startup company is a daunting task even for the most seasoned entrepreneur, and often overwhelming to academic researchers who wish to translate their ideas. Many investigators fall back on the strength of their research and the corresponding product, expecting that companies will spring up around the product. As discussed above, most pre-clinical research has a long path to travel to reach clinical trials and requires a talented and dedicated entrepreneurial team. Often, the lead investigators of these teams do not wish to give up control of their research. However, academic investigators are rarely motivated to ensure the success of a start-up; already having established careers, well-paying jobs, and many conflicting responsibilities. In these cases, the investigators are better positioned to be Chief Technology Officers or members of the company's advisory board. From there, the investigator is still positioned to give input to the burgeoning company without changing their career path. Often underutilized resources that can greatly benefit a company are post-doctoral students and recently graduated Ph.D. students. At that start of their career, these trainees do not have as many pressures and demands upon their time, and they are intimately familiar with the potential project. They are uniquely positioned to lead the spinout team but should be counterbalanced with an experienced team member. It is essential for all researchers contemplating starting a biotechnology company to understand their own limitations. Rarely do researchers have the necessary background to fill executive positions within the startup. Outside hiring for positions such as the Chief Executive Officer and consulting firms could make the difference between success and failure, regardless of the market strength of the product.

### Intellectual Property

The core of any technology-based startup is its intellectual property and patent protection. Patents allow startup companies to maintain a competitive advantage over entrenched competitors as the startup grows. Patentable technology arising from any academic institution or federally funded research falls under the Bayh-Dole Act, passed in 1980. Within the act, the federal government retains several rights but assigns ownership to the institution that the inventor works for (Stevens, 2004). It also obligates universities to pursue patents on any patentable federally funded technology, which has led to the necessity of patent attorneys on university staff, often in the form of a technology transfer office. Despite the wording of the law, many patentable technologies remain unpatented by universities due to issues with the prior art. To patent an invention in the United States, the invention must be considered significantly novel, useful, and non-obvious^3^. To be novel, the TEMP must be significantly different from previous inventions, denoted as prior art, which includes other patents and other information considered public domain knowledge. Investigators run afoul of this requirement when they publish their invention before patenting. Published research articles, conference posters, and even scientific data presentations are considered to be part of describing the invention into the public, creating an obstacle to patenting. However, this does not mean investigators cannot publish their work if they intend to file a patent, but careful consideration should be given to the timeline of publication to allow time for patent filing. Consultation with their intuitions patent attorneys early and often is the best way for investigators to avoid any patenting issues and to ensure a successful filing.

Given the current globalization of medical affairs, startups should also be looking at international markets. Patenting laws will vary by country, but any patenting within the United States will start a countdown of a year, after which the ability to pursue a patent in other countries will become limited. As one of the largest markets for TEMPs, Europe represents a key area for biotechnology companies to file for patents. It is possible to file in the individual countries, each with their own rules and processes, but it is cheaper to file under the European Patent Convention (EPC). Under the EPC, TEMP patents have similar requirements to the U.S. patent process, namely novelty, the inventive step, and industrial applicability (Belda et al., 2014). Patents filed under the EPC are considered by the European Patent Office (EPO). Unlike the U.S., the EPO is concerned with the ethics of the patented product. TEMPs utilizing human embryonic tissue, cloning of humans, or modifying the germ line genetic identity is un-patentable under the EPC^4^. Another large market, China, offers patents through the Chinese National Intellectual Property Administration. Chinese patent law has similar requirements of novelty, inventive step, and industrial applicability. Each country will require time and resources in order to file. Therefore, inventors should take time to define critical markets that they may wish to enter and patent accordingly. The bed to bedside and back again approach eases this process because potential markets and revenue streams can already be defined.

### Key Partners and Sources of Funding

Hospitals have well-established drug and device-based therapy control centers but lack the technical and logistical infrastructure to support TEMPs. Thus, a key challenge for the development of innovative therapies is how it will be adopted and implemented in existing clinical practice (Gardner and Webster, 2016). Access to established cell-manufacturing facilities and close alliances between the scientists and clinicians is essential to navigating this obstacle. The decision to partner with an established company that already markets TEMPs may facilitate clinical integration through access to their facilities, expertise in the product market sector, and an existing clientele base. The decision to partner with an existing company or establish a start-up company both carry unique challenges. Developing a new company requires significant effort to receive buy-in from investors or venture capital funding. In this route of clinical translation, the hire of a consultant is a common and recommended practice. Consultants can offer keen insight into navigating regulatory pathways and market infiltration, often with many years of expertise in several professions. When hiring, a consultant contract should be drafted with specific and definitive language to ensure no confusion over the expectations, inclusions, and exclusions of the agreement. The Technology Transfer Office can also be a valuable assistant for product translation. With a concerted effort to apportion technologies, knowledge, and facilities between institutions, the Technology Transfer Office aims to ensure accessibility and dissemination of new scientific developments to a wide range of users (Lu et al., 2015).

The associated costs of bringing a TEMP to market can be quite high and may demand several rounds of financing, in addition, to support in development from investors. Securing investors can be more difficult for TEMPs; often people hesitate to invest in regenerative medicine technologies due to a lack of clarity on regulatory pathways, clinical translation, and reimbursement (Bertram et al., 2012). To ameliorate investor opinion, the most important factors in securing investments are clarity in value proposition and proof of concept.

Funding allocation is the most direct form of translational support, often granted with the guarantee of a return. An intermediate form of return or reassurance takes the form of peer-reviewed publications. Specifically, financial risk can be reduced by the successful demonstration of clinically relevant TEMPs against clinical standards in an academic setting through the publication of their technology in high-profile journals. Robust evidence of proof of concept, through high quality measurable clinical data, guarantees a strong device history file (Hollister and Murphy, 2011). High-impact papers can be very beneficial to maintaining and securing scientific and investment partners, and from which strong patents can evolve naturally. As the product is further developed, clinical trials must be carefully constructed to validate efficacy and safety. While developing a repository of strong academic and clinical data, a successful company must maintain financial security, design a feasible path toward translation, and have partners who champion the technology.

### The Current State of the TEMP Industry

The TEMP field has a long history of companies that failed to reach commercial success. Since its beginnings in the 1990s, the TEMPs industry has been through many ups and downs as new high-profile companies fail to meet the scientific, regulatory, and public expectations set for them. In 2012, an in-depth analysis of the TEMP industry sector by Jaklenec et al. suggested that the industry has begun to stabilize (Jaklenec et al., 2012). The regenerative medicine market is expected to grow from $10.8 billion in 2016 to over $22 billion in 2025 worldwide^5^. This burgeoning market represents the numerous companies that are entering the commercial phase of their product development. Kim et al. identified 49 tissue engineering companies active in the United States, 21 of which reported an estimated 9 billion dollars in sales for tissue engineering-related products for the year of 2017 (Kim et al., 2019). Of those companies, most focus on point-of-care technology utilizing autologous cell treatments. Currently, the market has accepted simplified autologous cell therapy products, but many companies are moving forward with more complex allogeneic therapies, and their fate will, in part, be determined by the regulatory approval process.

## Governmental Oversight

### Food and Drug Administration (FDA)

Regulation represents one of the most significant hurdles for TEMPs. Eighty six percent of all clinical trials fail to get FDA approval and reach a clinical setting, resulting in significant economic burdens (Wong et al., 2018). For TEMP startups, failure to get FDA approval could result in the demise of the company, while FDA trials create a significant hurdle for any competitor wishing to enter the market. Successful regulatory strategy starts before the product is ever presented in the office at the FDA, and investigators should be aware of the potential regulatory path that their invention could take in order to ease the path further down the road. Compared to European Union regulation, the review process within the FDA is often complex and is dictated by the center within the FDA that is responsible for the TEMP.

### The Office of Combination Products

**Figure 2:**
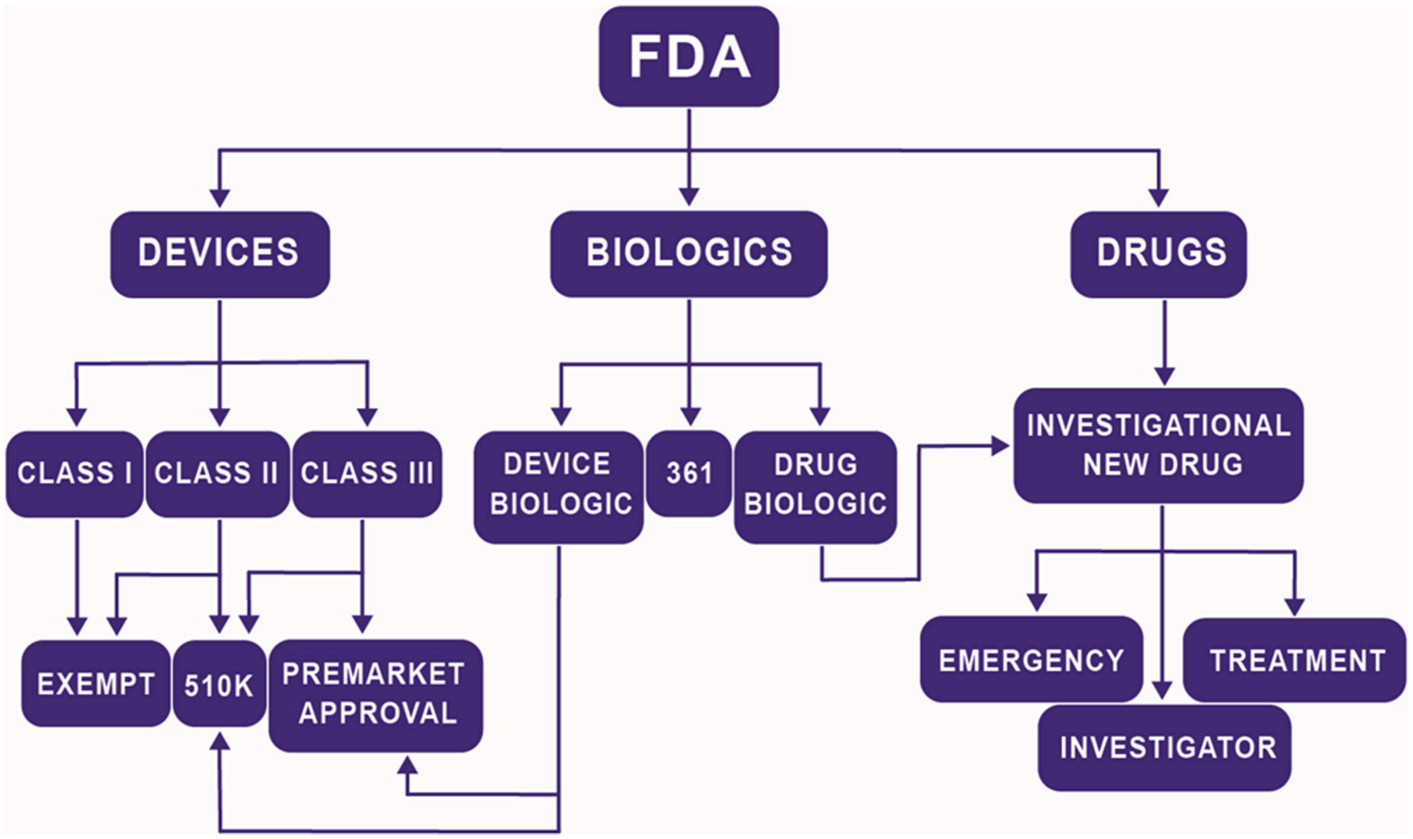
Simplified flow chart of the path a TEMP will follow to determine regulatory path at the FDA.

Navigating the multiple centers and divisions within the FDA is difficult, especially for TEMPs. The FDA rarely dictates to a company the tests they must perform in order to prove that their product is safe. Instead, it is a collaborative discussion where the company is responsible for formulating a regulation plan, and the FDA either approves of the plan or recommends changes. The structure and intensity of that plan can vary widely by product but is heavily influenced by the center under which the product falls. The three major centers at the FDA related to most TEMPs are the Center for Devices and Radiological Health (CDRH), the Center for Biologics Evaluation and Research (CBER), and the Center for Drug Evaluation and Research (CDER). In the past, a medical product would fall under one of three possible classifications: a device, a biologic, or a drug, and be sent to the appropriate center. With the advent of TEMPs, it is no longer as easy of a task to make those distinctions. Now, a product may be a novel scaffold that releases a drug at a controlled rate or a combination therapy that includes both stem cells and drug treatment. The Office of Combination Products (OCP) was founded to help solve this problem by offering guidance to companies on determining which center they should approach for approval. The critical consideration for investigators is understanding that choice of Center for regulation can significantly affect the time to market. On average, devices are the quickest to be approved (~6 years) while drugs take the longest (~11 years) and biologics fall in between (~9 years) (Naghshineh et al., 2014). These are not strict rules but is an illustration of how different classifications can affect the overall health of the startup. In general, the highest risk component of the product determines its regulatory pathway. Figure 2 illustrates a generalized flow chart of the path a TEMP will follow. Considerations should be made at the bench to what is already approved and how is the current product going to be approved to decrease the resources required to get FDA approval.

### Center for Devices and Radiological Health

Products defined as devices have arguably the most direct approval process. Class I devices are considered the lowest risk. In general, these devices are of simple design, such as tongue depressors. Seventy four percent of class I devices follow the exempt pathway which does not require premarket notification or approval. There are very few TEMPs that fall into the classification as Class I. Class II products are more complicated devices but are typically non-implantable, non-invasive, and non-significant risk. A few class II devices will fall under exempt status, but the majority follow the premarket notification, 510(k) pathway. The 510(k) pathway requires proving that the device is similar in usage, risk, and efficacy to products available before 1976. The final classification is a class III device, which constitutes products of the highest risk. These devices require premarket approval, the most stringent regulation process in the CDRH and include preclinical and clinical trials. There has been a recent trend toward companies attempting to utilize the 510(k) pathway to avoid the burden of clinical trials (Van Norman, 2016b). The most effective way for TEMP devices to utilize this pathway is through the use of pre-approved components when available. Simplification of the product also helps by minimizing risk and concerns over component interaction.

### Center for Drug Evaluation and Research

A limited number of TEMPs are directed toward the CDER, which will require the most stringent review process. CDER governs their regulatory process through the investigational new drug pathway which has three classifications; investigator, treatment, and emergency. A majority of products will fall under investigator classification, which allows new drugs or drugs with new indications to be studied through clinical trials. Treatment classifications are for drugs which treat a small population that cannot be investigated through the normal pathway. Emergency classification options are for drugs that require approval in a time frame that is unrealistic using the standard method. The investigational new drug pathway requires clinical trials, which results in an often-protracted time difference between CDER and the other two centers. The cost of conducting clinical trials and the low success rate is prohibitive to startup companies and limits the ability for a single product to spin out of a research lab. However, there are paths investigators can take to make sure their TEMP reaches the clinical market. Strategic partnerships with large companies can provide investigators the resources needed to get through clinical trials. Established companies will have different customer requirements, such as target customer segments, market /product life cycles, and competition with previously approved products. Investigators should critically evaluate what effects their research will have on their future market and whether an established company would welcome a new product. Startup companies have options as well. During the initial investigation, small markets may not seem appealing for entrepreneurs. However, treatment new drug pathways make it more feasible to enter the smaller markets first. Those markets can then pay for future clinical trials and provide data for investors.

### Center for Biologics Evaluation and Research

Products composed of living tissues or products potentially derived from living tissues are defined as biologics and are regulated by the CBER^6^. Human tissues or cells that are minimally manipulated and meant for homologous use are referred to as “361” and do not need to follow the premarket approval, 510(k) pathway, investigational new drugs approval, or a biologics license application. Human tissues that do not fall under the 361 designation face a potentially complicated and extensive regulatory pathway. The biologics regulatory process is a hybrid of the device and drug regulatory processes. Device biologics follow the 510(k) and premarket approval process similar to devices but with additional biologic related regulations. Certain biologics, such as vaccines, follow the investigational new drug pathway. A biologics license application is required for any manufacturers of biological products and covers the manufacturing process and medical effects of the product. Human tissues face special scrutiny, especially with the fears over transmittable diseases. Investigators should consider the source of the tissue and its original function. Matching the function between the source and the product can ease regulatory concerns and quicken the review process. Xenograft tissues that serve the same functions in TEMPs products fall under device biologics and may undergo a simplified regulatory pathway. Autologous vs. allogeneic is often a crucial choice for new TEMPs. From, an industry perspective, sourcing the product from allogeneic tissues allows for decrease production costs and improved quality assurance. Autologous tissues have the benefits of reduced regulation and decreased rejection concerns. Deciding between components can and should occur during bench-top research. Switching components before the inception of a human clinical trial is challenging due to the need to possibly generate new preclinical trial data.

### Simplifying the Product

The pressures of survival in academia, such as publishing, can lead investigators to add components to their inventions that have incremental benefits but allow their research product to be innovative. Although this approach can lead to an improved product in the clinic, it can have added unnecessary complications to the regulatory FDA approval process. Additionally, the FDA works on precedent, comparing new products to those that have been approved previously. The most direct way to move through the FDA and reach the market is to be able to compare some or all of the product to previously approved products. Each added component or incremental change will be scrutinized and can result in additional time and testing prior to approval. Investigators need to weigh the benefits of including a new component with the added resources needed to justify its presence. Not every minor improvement should be implemented. Deciding on what to components to include after benchtop studies is problematic because it is often too late and too expensive to make the changes even for established companies. By looking ahead and planning for the regulatory process, investigators can eliminate potential research options and focus on the key components that deliver the most value.

## European Medicine Agency (EMA)

There are many similarities drawn between the EMA and the FDA, however, the FDA is often considered slow and very risk-averse. On the reverse side, the EMA regulatory process is often considered too quick to approve (Van Norman, 2016a). Medicines based on genes, cells, and tissues are regulated by the same pathway and are termed as advanced therapy medicinal products (ATMPs) by EMA. They are broadly classified as gene therapy medicines (GTM), somatic cell therapy medicines (SCTM), and TEMPs (Yu et al., 2018). SCTMPs and TEMPs are distinguished on the basis of their mechanism of action. In the case of SCTMPs, cells or tissues exert a pharmacological action, whereas TEMPs are intended for tissue regeneration and repair (Izeta et al., 2016). ATMPs, when combined with medical devices, are categorized as combined ATMPs (Chabannon et al., 2014). A benefit of the EMA regulation of ATMPs is they all follow a similar regulatory process governed by the Committee for Advanced Therapies (CAT). The legal framework “Regulation (EC) 1394/2007” for tissue-engineered products was established in the European Union (EU) in 2007 (Pellegrini et al., 2018).

### Research and Development

The EMA formally supports research and development of ATMPs by providing “scientific advice and protocol assistance” to developers. This program provides insight and guidance on the necessary tests and studies required for the development of ATMPs. Additionally, researchers can also consult EMA to validate whether their product qualifies as an ATMP before applying for market authorization^7^.

EMA received 298 queries for ATMP classification and 293 requests for scientific advice until May 2018^8^. During this period over 500 ATMP based clinical trials were initiated in the EU, and ~25% of those trials were done to test TEMPs (Boran et al., 2017). Clinical trials have been conducted for a wide range of pathophysiological conditions using ATMPs; however oncological, musculoskeletal, cardiovascular and immunological disease appear to be the priority areas (Boran et al., 2017).

### Market Authorization (MA)

The CAT is officially designated by EMA for the assessment of “market authorization” (MA) applications. Moreover, the CAT is also responsible for developing new guidelines for the classification of ATMPs, which are published monthly on EMA's website. The application for MA begins with the submission of a comprehensive dossier to CAT that provides details of the product to be reviewed. Scientific specialists at CAT review the quality, safety, and efficacy of ATMPs and prepare a draft opinion based on the non-clinical and clinical data provided by the developers (Salmikangas et al., 2015). Until February 2019, the CAT had received a total of 22 MA applications, out of which 13 applications received a positive draft, and 4 received a negative draft, whereas 5 applications were withdrawn^9^. The CAT submits it's draft to the Committee for Medicinal Products for human use (CHMP) for adoption of final opinion on MA. After authorization EMA continues to monitor the ATMPs in the market through its post-marketing surveillance program to ensure patient safety (Celis et al., 2015).

### Commercialization of ATMPSs

The EMA has granted a total of 10 ATMPs market authorization. ChondroCelect® (TiGenix, Belgium) a cartilage cell-based product, was the first ATMP to be approved in 2009, followed by a gene therapy product called Glybera® (Uniqure, Netherlands) in 2012. Holoclar® (Chiesi, Italy) became the first stem cell therapy to be approved by EMA in 2015 (Pellegrini et al., 2018) and in the following years more stem cell-based medicines including Strimvelis™ (GSK, UK) and Alofisel® (TiGenix, Belgium) have been marketed (Yu et al., 2018). To-date 4 ATMPs have been withdrawn from the market; these include CondroCelect®, Glybera®, and Provenge® (MolMed, Italy), due to poor commercial performance (Abou-El-Enein et al., 2016), whereas the approval of MACI® (Vericel, US) was suspended by the EMA due to the closure of the EU manufacturing facility (Boran et al., 2017).

The EMA has made extensive efforts to encourage manufacturers to develop ATMPs in the past decade, yet the number of approved ATMPs remains considerably low. Furthermore, the clinical promise of the approved ATMPs has not translated in to commercial success. The major impediments in the success of ATMPs in the EU include; high development cost, complex regulatory procedures, lack of efficient pricing and reimbursement schemes, limited target population, and potential risk associated with the use of ATMPs especially gene therapy (Abou-El-Enein et al., 2016; Barkholt et al., 2018; Yu et al., 2018).

Moving forward potential ATMP developers ought to take more advantage of the EMA's guidance programs for ATMP development. Early efforts shall be made to ensure that the product under development categorizes as an ATMP. Once the product category has been determined manufacturers can seek help from EMA to ascertain the quality standards (purity, stability, etc.) required for the development of ATMPs (Barkholt et al., 2018). During the developmental phase of the product, manufacturers may request certification of quality and non-clinical data to ensure that they are working in the right direction to obtain market authorization^10^. Adherence to these critical steps will significantly alleviate the risk of products fading out in infancy and facing MA rejections.

## Dermagraft

Despite optimism for the future of the TEMP market, it is important to note that the hurdles that caused so many companies to fail still exist. Consider Dermagraft®, a tissue engineered dermal skin product currently offered by Organogenesis. Dermagraft is one of the most infamous tissue engineered products currently on the market, due to failures of Advance Tissue Sciences Inc. (ATS), Smith & Nephew Plc., and Shire Plc. to make Dermagraft profitable despite significant investment. Pangarkar et al. published a case study of ATS in 2010, highlighting several of the key concerns discussed in this article (Pangarkar et al., 2010). Dermagraft is a potential dermal skin replacement, created by dermal fibroblasts grown on a 3D scaffold. By all accounts, Dermagraft was a product that worked, particularly for diabetic foot ulcers (Marston et al., 2003). The business side of the product, however, proved to be much more challenging. From the beginning, the owners of Dermagraft faced issues with governmental approval, overestimation of market potential, and market penetration. Clinical trials of Dermagraft started in 1991, with expectations for FDA approval by 1995. It would not be until 2001 that ATS would receive FDA approval of Dermagraft for diabetic foot ulcers. The delay in FDA approval can be contributed to many different controversial aspects of the approval process, however, it is fair to say there was confusion and uncertainty on both the parts of the FDA and ATS on how regulation of TEMPs should be handled. For example, in 1998, the FDA requested additional clinical trials for Dermagraft, despite an expert advisory committee recommending the product for approval (Pangarkar et al., 2010). In subsequent clinical trials, ATS changed their testing parameters, leading to contention with the FDA over the success of the trial (Pangarkar et al., 2010). Regulation of TEMPs within the FDA and worldwide has significantly progressed since the early 2000s. However, the same arguments over our understanding of how TEMPs interact with the human body still plague the review process and can lead to significant delays in approval and could impose a sizeable financial burden on startups. While seeking FDA approval for Dermagraft, ATS pursued many different other potential TEMPs. These products included Skin^2^® (used to screen cosmetic pharmaceuticals), heart valves, artificial grown livers, and growing cartilage. This pipeline involved a $17 million annual R&D budget, of which ATS saw little return due to multiple failed products. At the same time, revenue from the few successful products failed to meet projections that ATS had set for them. After FDA approval for Dertmagraft in 2001, ATS projected only $7.5 million in revenue in 2002, and $12 million in 2003, much lower than the initial projections used for the original justification of the R&D budget (Pangarkar et al., 2010). This mismatch in sales emphasizes the need to understand and plan for the low market penetration that is experienced by many TEMPs. As discussed previously, health care systems are slow to adopt the radical changes inherent to TEMPs, which can lead to overestimations of market potential and over promising to investors. Lower, more realistic market projections can protect companies from investment scares.

In 2002, ATS would sell Dermagraft (with other ATS products) to their marketing partner, Smith & Nephew, for $12 million as part of their chapter 11 filing. Smith & Nephew would sell Dermagraft and TransCyte® to Advanced Bio-Healing in 2007 for an undisclosed amount. In 2014, Organogenesis purchased Dermagraft for $300 million from Shire, who in turn had acquired Advanced Bio-Healing for $750 million in 2011 (Fikes, 2014). Despite achieving FDA approval and showing clinical benefits, Dermagraft has failed again and again to turn a profit, revealing how much deeper and more complicated developing a TEMP can be. One potential reason for the failure of Dermagraft is its price. Dermagraft currently averages about $1,500 per 2″ × 3″ pad, which is incredibly high when compared with the standard bandaging used in foot ulcer treatment. In addition to higher pricing, TEMPs are often not covered by health insurance and Medicare. Shire reportedly blamed changes in federal Medicare coverage of wound-healing products on their decision to sell Dermagraft (Fikes, 2014). As a more recently developed field, TEMPs can have difficulty proving their long-term cost benefits to health coverage providers, which in turn can affect their market penetration. This issue can be traced back to investigators, who often fail to account for the potential manufacturing repercussions during the benchtop decisions. Ironically, Organogenesis (a one-time competitor of ATS) still offers Dermagraft. Investigators should take this as a sign that health care is warming to the idea of TEMPs.

## Conclusions

The path from the bench to human application is potentially long with many unexpected turns and potentially full of minefields, where each decision can have far-reaching consequences. In order to increase the likelihood of clinical success, investigators need to look beyond the benchtop and consider the direction their research will follow and allow those considerations to guide and shape their research plans. This approach includes identifying and developing a clear understanding of a clinical need and establishing a research program to address that human need. Bedside to bench and back again not only increases the likelihood for the successful transition to the clinic but also impacts benchtop research. By understanding how their research can fit into the future, investigators can develop effective impact statements, increased the relevance and applicability of their research, and develop strategic partnerships. By moving beyond scientific constraints that prevent clinical translation of TEMPs, investigators can develop strategies for developing successful technologies.

## Author Contributions

BO was involved in the conception, writing, drafting, and editing of this article. CI was involved in the writing and figure making of this article. OM was involved in the writing of this article. BB was involved in the conception and editing of this article.

### Conflict of Interest Statement

The authors declare that the research was conducted in the absence of any commercial or financial relationships that could be construed as a potential conflict of interest.

## Footnotes

^1^https://report.nih.gov/categorical_spending.aspx

^2^https://venturewell.org/i-corps/

^3^https://www.uspto.gov/patents-getting-started/general-information-concerning-patents

^4^https://www.epo.org/law-practice/legal-texts/html/epc/2016/e/r28.html

^5^https://www.statista.com/statistics/871252/regenerative-medicines-market-value-worldwide-by-therapy-type/

^6^https://www.fda.gov/BiologicsBloodVaccines/default.htm

^7^https://www.ema.europa.eu/en/human-regulatory/research-development/advanced-therapies/support-advanced-therapy-developers

^8^https://www.ema.europa.eu/en/documents/presentation/presentation-advanced-therapy-medicinal-products-atmps-atmp-regulation-patrick-celis_en.pdf

^9^https://www.ema.europa.eu/en/documents/committee-report/cat-monthly-report-application-procedures-guidelines-related-documents-advanced-therapies-february_en-5.pdf

^10^https://www.ema.europa.eu/en/documents/presentation/presentation-advanced-therapy-medicinal-products-atmps-atmp-regulation-patrick-celis_en.pdf
